# Supplementary material for: Clade distillation for genome-wide association studies
Source: Genetics. 2025 Aug 7;232(1):iyaf158. doi: 10.1093/genetics/iyaf158 (PMC12667359; doi:10.1093/genetics/iyaf158)
Supplement: iyaf158_Supplementary_Data [file iyaf158_supplementary_data.pdf]

Supplementary Materials for
“Clade Distillation for Genome-wide Association Studies”

Ryan Christ, Xinxin Wang, Louis J.M. Aslett, David Steinsaltz, & Ira Hall

August 6, 2025

1 Supplementary Figures

1.1 Checkpointing

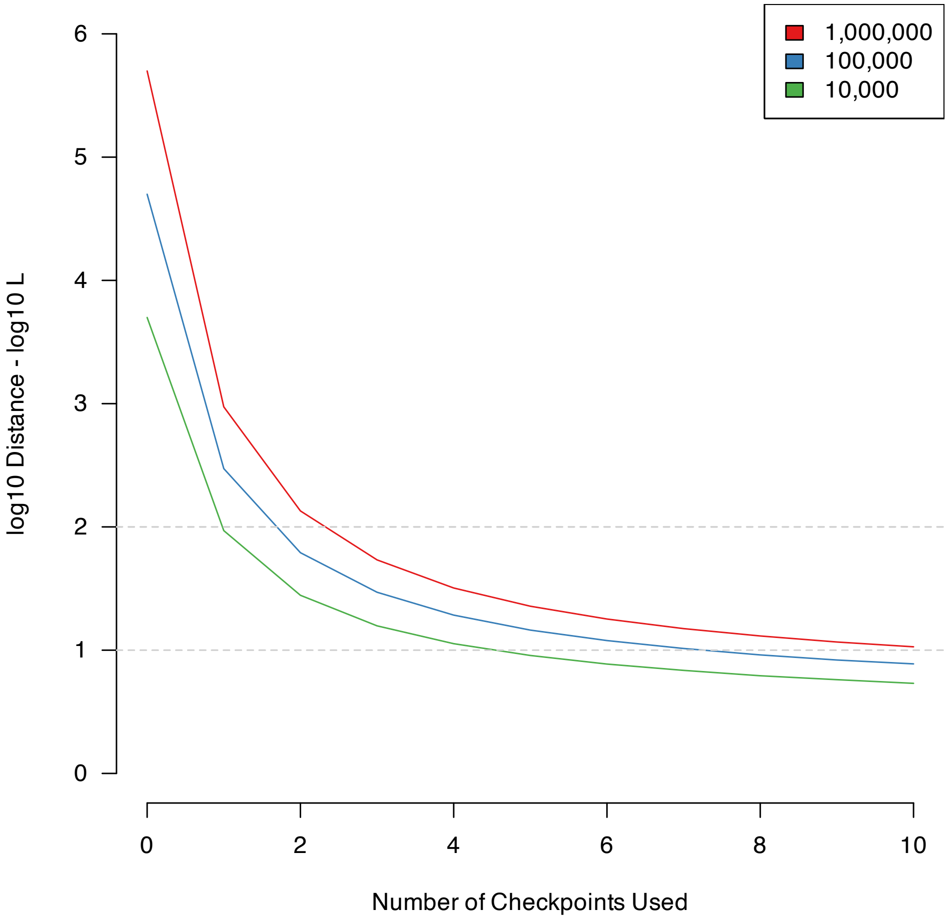

Figure 1: **Computational Cost Using Optimal Checkpointing.** We consider the total number of variants the forward algorithm needs to iterate over (the total distance D it has to cover) in order to visit all loci on a chromosome of length L using our optimal checkpointing strategy. We plot the $\log_{10}(D/L)$ as a function of the number of checkpoints, C, available for chromosomes of three potential lengths indicated by the legend: $L \in \{10^4, 10^5, 10^6\}$. The dotted gray horizontal lines highlight that we are able to reduce D to under $100L$ with only 3 checkpoints and nearly $10L$ with only 10 checkpoints in all cases.

1.2 Null Simulation Q-Q Plots

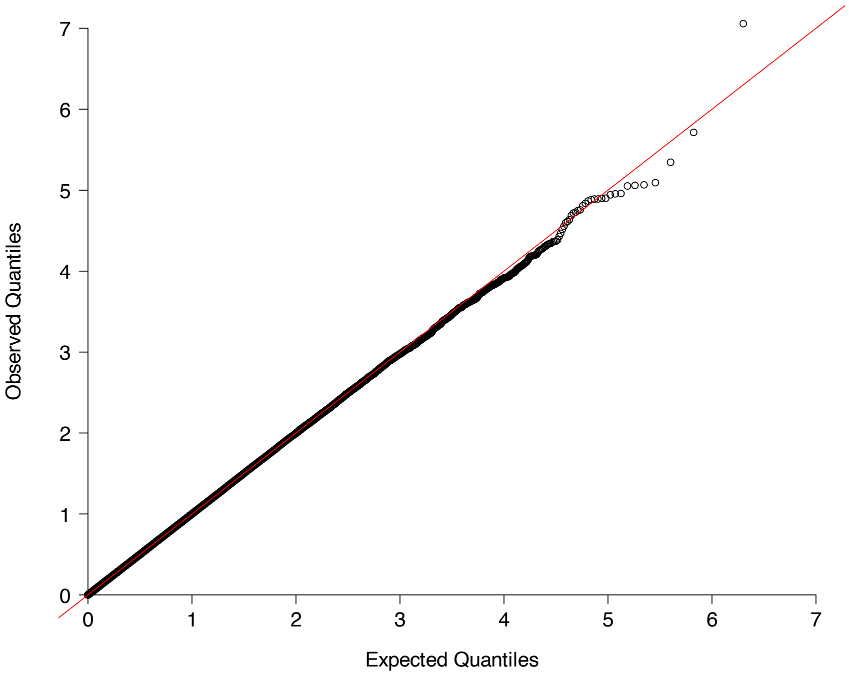

Figure 2: **SMT Subtest Q-Q plot.** Q-Q plot of samples of $-\log_{10}\left(p^{(\ell)}_{\text{SMT}}\right)$ based on 1 million independent phenotype vectors simulated under the null hypothesis.

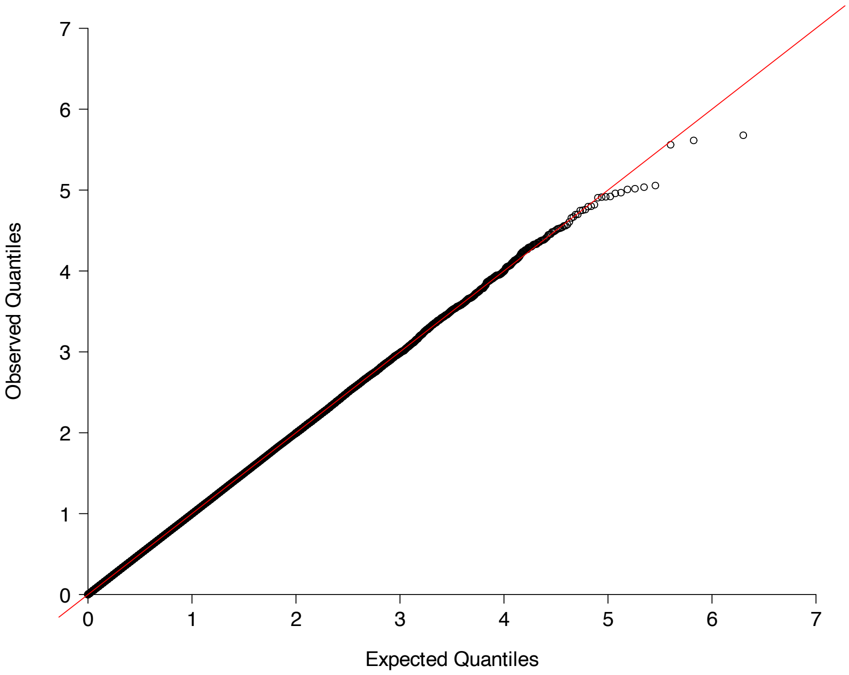

Figure 3: **SD Subtest Q-Q plot.** Q-Q plot of samples of $-\log_{10}\left(p^{(\ell)}_{\text{SD}}\right)$ based on 1 million independent phenotype vectors simulated under the null hypothesis.

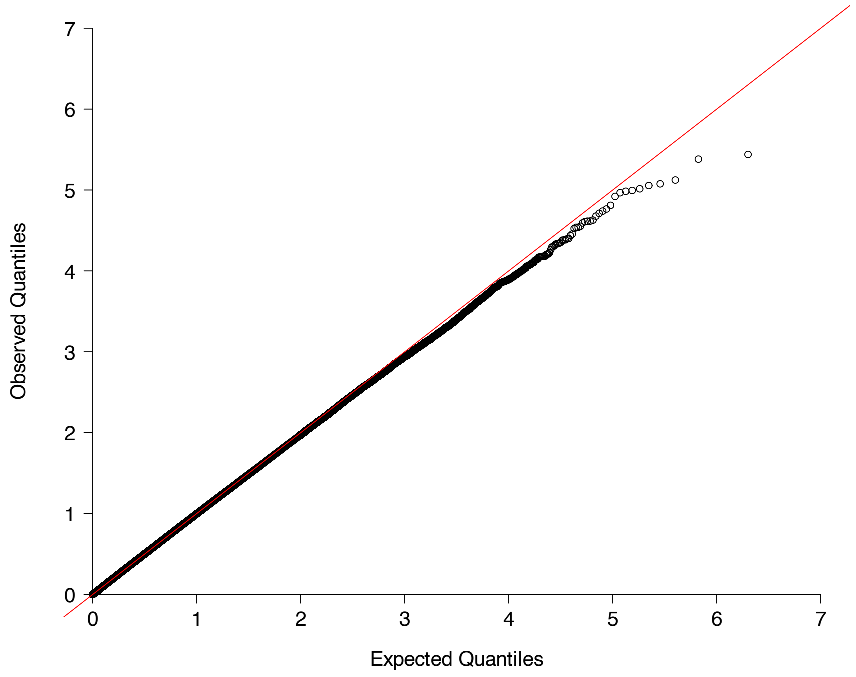

Figure 4: **QForm Subtest Q-Q plot.** Q-Q plot of samples of $-\log_{10}\left(p_{\mathrm{Q}}^{(\ell)}\right)$ based on 1 million independent phenotype vectors simulated under the null hypothesis.

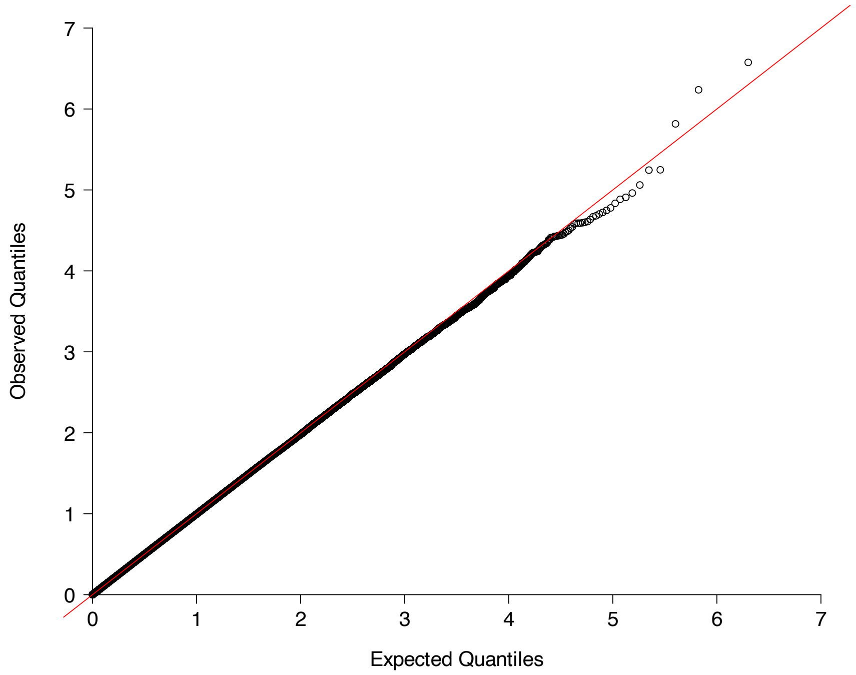

Figure 5: **LOCATER Combined Q-Q plot.** Q-Q plot of samples of $-\log_{10}\left(p_{C}^{(\ell)}\right)$ based on 1 million independent phenotype vectors simulated under the null hypothesis.

1.3 Power Simulation Effect Sizes

As outlined in the Phenotype Simulation subsection of the Methods in the main text, we simulate phenotypes under the model

$$Y \sim A\alpha + X_{\mathcal{A}}\beta + \epsilon \tag{1}$$

where $X_{\mathcal{A}} \in \{0,1,2\}^{n\times a}$ is a matrix of genotypes corresponding to the $a = |\mathcal{A}|$ causal variants and $\epsilon \sim N(0, I_n)$. The precise effect sizes induced by our simulation scheme for any specific $X_{\mathcal{A}}$ will be affected by the LD structure among those variants and the precise genotypes counts for each variant. Here we provide a rough estimate of the magnitude of the causal variant effect sizes β_js induced as a function of the total association signal strength (s) and the minor allele count, which we denote by m in this section, by making a few simplifying assumptions. For a given causal variant with genotype vector X_j, the the contribution of that genotype to $\mathbb{E}[Y]$ is

$$\beta X_j = \beta \|X_j\| \frac{X_j}{\|X_j\|} = \beta^\star \frac{X_j}{\|X_j\|}. \tag{2}$$

We will call $|\beta_j|$ the absolute effect size and $\beta^\star = |\beta_j| \, \|X_j\|$ the standardized absolute effect size.

We make the following assumptions. First, we ignore LD among the causal variants. More precisely, assuming that $X_{\mathcal{A}}^\top X_{\mathcal{A}}$ to be a diagonal matrix. Since causal variants are chosen at random within a window in each simulation, the expected pairwise LD between variants is close to zero, especially among rare variants. Second, we ignore migration and assume Hardy-Weinberg equilibrium (HWE). Recall that under the three population demographic model we use in our main power simulations, we draw 10,000 samples from each of three 1000 Genomes populations (YRI, CHB, and CEU). For each causal variant, we assume that minor alleles are only found in one of those three populations (ignoring migration) and that the genotypes for each causal variant are observed at HWE proportions within that population.

Under these assumptions, we will use $\widetilde{\beta}(s,a,m)$ denote our estimate of the absolute value effect size $|\beta_j|$ assigned to a causal variant j with minor allele count m in a simulation with a causal variants and a desired total association signal strength s. Recall we sample $N^\star = 20,000$ haplotypes from each of our three simulated sub-populations. Under our assumption of HWE and ignoring migration, after projecting out the population membership indicators, a genotype with minor allele count m has a genotype vector length $\|X_j\|$ of

$$l(m) = \sqrt{N^\star \left(\tfrac{m}{N^\star}\right)\left(1 - \left(\tfrac{m}{N^\star}\right)\right)\left(1 - \tfrac{1}{N^\star} - \tfrac{m}{N^\star(N^\star-1)}\right)}. \tag{3}$$

The last term arises as a finite sample correction in the following variance calculation, assuming HWE. Let $p^\star = m/N^\star$ and $X_{ij} = G_1 + G_2$ where $G_1, G_2 \sim \text{Bern}(p^\star)$.

$$\begin{aligned}
\mathbb{E}\left[\|X_j\|^2\right] &= \frac{N^\star}{2}\text{Var}[X_{ij}] = \frac{N^\star}{2}\left(\text{Var}[G_1] + \text{Var}[G_2] + 2\text{Cov}[G_1, G_2]\right) & (4)\\
&= N^\star\left(p^\star(1-p^\star) + \mathbb{E}\left[\mathbb{E}\left[(G_1 - p^\star)(G_2 - p^\star)\,|\, G_1\right]\right]\right) & (5)\\
&= N^\star\left(p^\star(1-p^\star) + (1-p^\star)\left(\frac{m-1}{N^\star} - p^\star\right)p^\star + (-p^\star)\left(\frac{m}{N^\star - 1} - p^\star\right)(1-p^\star)\right) & (6)\\
&= N^\star p^\star(1-p^\star)\left(1 - \tfrac{1}{N^\star} - \tfrac{p^\star}{(N^\star-1)}\right) & (7)
\end{aligned}$$

Plugging this this estimate of $\|X_j\|$ into Equation (2) and rearranging, we have

$$\widetilde{\beta}(s,a,m) = \beta^\star(s,a)\left(l(m)\right)^{-1}, \tag{8}$$

where $\beta^\star(s,a)$ is an estimate of the standardized absolute effect size. Recall that we define the total association signal strength s as the $-\log_{10}$ p-value that one would obtain by testing the resulting Y with an oracle ANOVA model that "knows" the causal variants. Ignoring LD, the oracle ANOVA model p-value is given by the right tail probability $1 - F_{\chi^2_a}\left(\sum_{i=1}^{a}\left(\beta^\star(s,a)\right)^2\right)$ where $F_{\chi^2_a}$ is the CDF of a χ^2 distribution with a degrees of freedom. Inverting this formula yields

$$\beta^\star(s,a) = \sqrt{\tfrac{1}{a}F^{-1}_{\chi^2_a}\left(1 - 10^{-s}\right)}. \tag{9}$$

In Figure 6 we plot our estimate of the inverse genotype vector length, $(l(m))^{-1}$, as a function of minor allele count m. In Figure 7, we plot $\beta^{\star}(s,a)$ as a function of s for the three values of a used in our simulations: 3, 9, and 15. As shown by Equation 8, our estimated absolute effect size $\widetilde{\beta}(s,a,m)$ can be written as a product of $\beta^{\star}(s,a)$ and $(l(m))^{-1}$. Table 1 provides $\widetilde{\beta}(s,a,m)$ for various combinations of s, a, and m relevant to our simulations. To get a sense of the signal-to-noise ratio of these effects, recall that these effect sizes are applied in the context of a noise term that is Gaussian with scale 1. Thus entries in Table 1 can be interpreted as estimates of Cohen's d: effect size divided by the standard deviation of the phenotype [1].

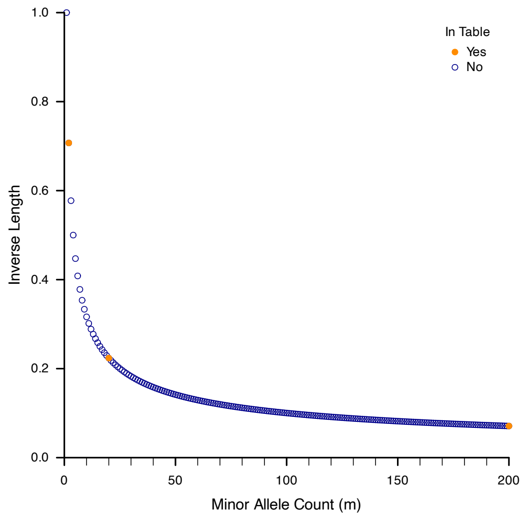

Figure 6: Estimated Inverse Length $(l(m))^{-1}$ as a function of minor allele count (m). Dots are displayed for all values of $m \in \{1, 2, \ldots, 200\}$. Dots corresponding to values of m explicitly calculated in Table 1 are highlighted in orange.

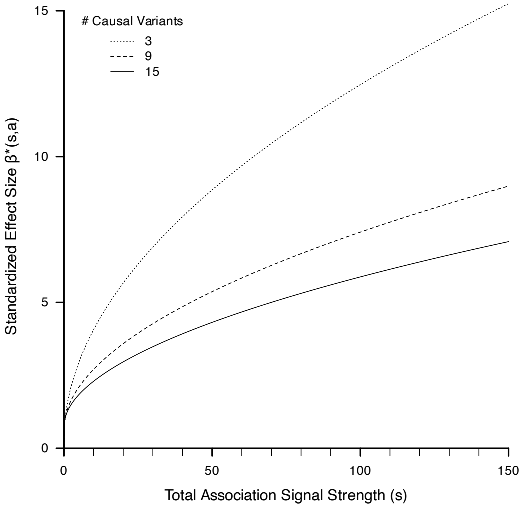

Figure 7: Standardized Effect Size, $\beta^{\star}(s)$, as a function of Total Association Signal Strength s.

**MAC**	**# Causal**	**Total Association Signal Strength (s)**												
**(m)**	**(a)**	10	20	30	40	50	60	70	80	90	100	110	120	130
2	3	2.87	4.01	4.88	5.61	6.26	6.85	7.39	7.89	8.37	8.82	9.24	9.65	10.04
2	9	1.91	2.54	3.02	3.43	3.80	4.13	4.43	4.72	4.99	5.24	5.48	5.71	5.94
2	15	1.62	2.10	2.46	2.78	3.05	3.30	3.54	3.76	3.96	4.15	4.34	4.52	4.69
20	3	0.91	1.27	1.54	1.78	1.98	2.17	2.34	2.50	2.65	2.79	2.92	3.05	3.18
20	9	0.61	0.80	0.96	1.09	1.20	1.31	1.40	1.49	1.58	1.66	1.73	1.81	1.88
20	15	0.51	0.66	0.78	0.88	0.97	1.05	1.12	1.19	1.25	1.31	1.37	1.43	1.48
200	3	0.29	0.40	0.49	0.56	0.63	0.69	0.74	0.79	0.84	0.89	0.93	0.97	1.01
200	9	0.19	0.25	0.30	0.34	0.38	0.41	0.45	0.47	0.50	0.53	0.55	0.57	0.60
200	15	0.16	0.21	0.25	0.28	0.31	0.33	0.36	0.38	0.40	0.42	0.44	0.45	0.47

Table 1: Estimates of the absolute value of effect sizes used in our simulations, $\widetilde{\beta}(s,a,m)$ as a function of m, a, and s. Minor allele counts should be interpreted as relative to a 20,000 alleles in a population; thus the estimates in this table correspond to variants with minor allele frequency 0.0001 ($m = 2$), 0.01 ($m = 20$), and 0.001 ($m = 200$).

1.4 Power, Source, & Localization Curves

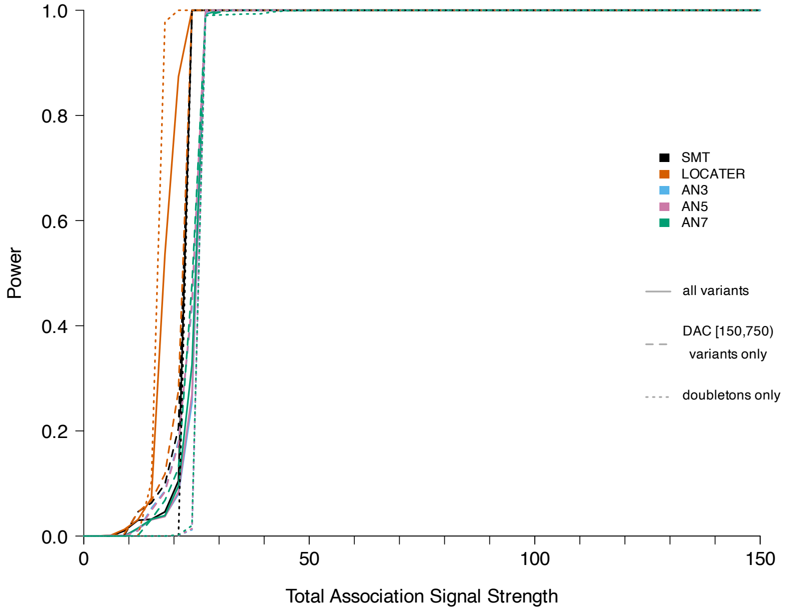

Figure 8: Power comparison between SMT, ARG-Needle, and LOCATER in simulations where there are 3 causal variants, all observed. Power is displayed as a function of total association signal strength s: the $-\log_{10}$ p-value that one would obtain by testing with an oracle ANOVA model that "knows" the causal variants.

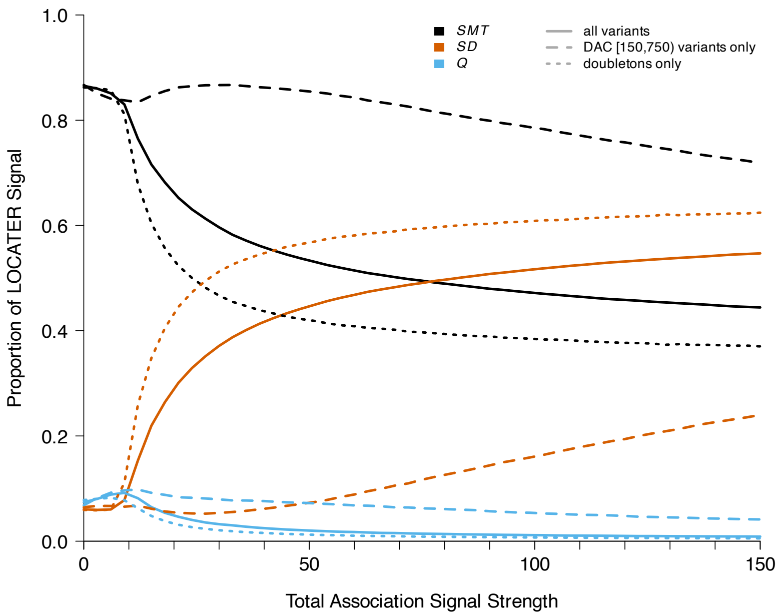

Figure 9: Average proportion of signal driven by each sub-test within LOCATER in simulations where there are 3 causal variants, all observed. For every simulation, the proportion of signal attributed to the SMT part of LOCATER (solid lines) defined as $\log\left(p^{\ell^\star}_{\mathrm{SMT}}\right) / \log\left(p^{\ell^\star}_{\mathrm{SMT}} p^{\ell^\star}_{\mathrm{SD}} p^{\ell^\star}_{\mathrm{Q}}\right)$ where $\ell^\star$ is the locus ℓ that achieves the largest combined LOCATER p-value. The proportion of signal attributed to SD (dashed lines) or quadratic form testing, abbreviated as Q (dotted lines), is defined analogously. Proportions are displayed as a function of total association signal strength s: the $-\log_{10}$ p-value that one would obtain by testing with an oracle ANOVA model that "knows" the causal variants.

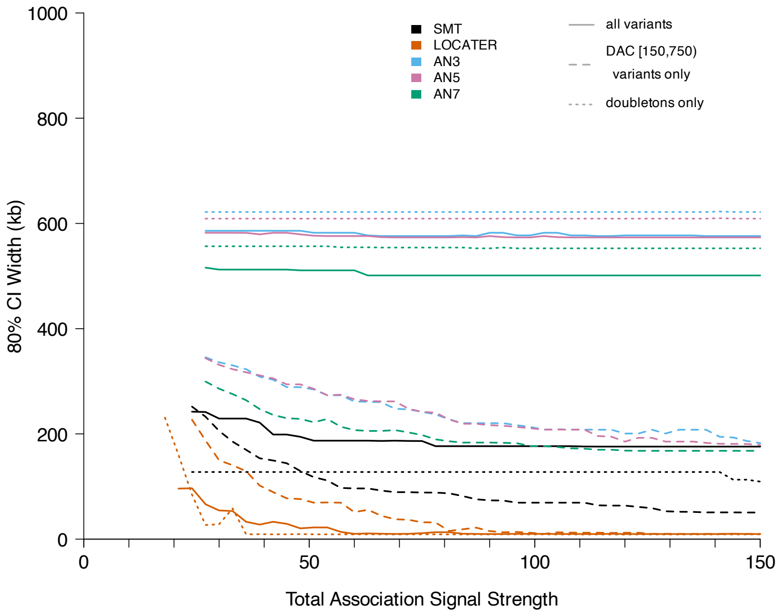

Figure 10: Width of confidence intervals (CI), centered on the position with the largest signal, that cover the midpoint of the causal region in 80% of simulations where there are 3 causal variants, all observed. In simulations where multiple variants are tied to have the largest association signal, we take the distance to the midpoint of the causal region to be the average distance from each of the tied variants. CI width is displayed as a function of total association signal strength s: the $-\log_{10}$ p-value that one would obtain by testing with an oracle ANOVA model that "knows" the causal variants.

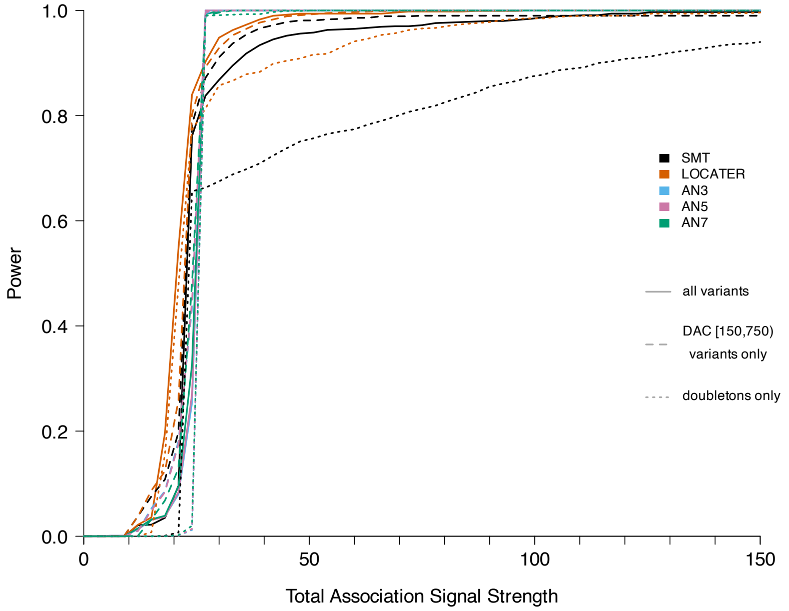

Figure 11: Power comparison between SMT, ARG-Needle, and LOCATER in simulations where there are 3 causal variants, all hidden. Power is displayed as a function of total association signal strength s: the $-\log_{10}$ p-value that one would obtain by testing with an oracle ANOVA model that "knows" the causal variants.

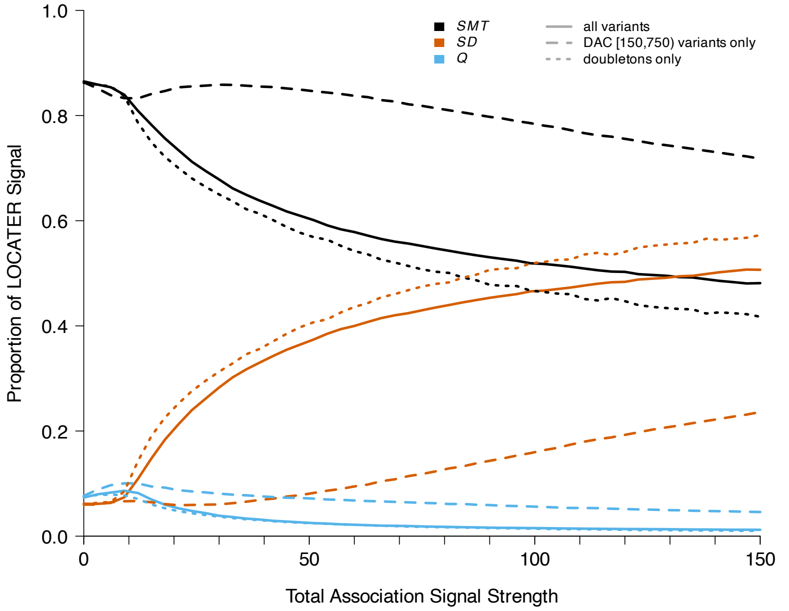

Figure 12: Average proportion of signal driven by each sub-test within LOCATER in simulations where there are 3 causal variants, all hidden. For every simulation, the proportion of signal attributed to the SMT part of LOCATER (solid lines) defined as $\log\left(p_{\text{SMT}}^{\ell^\star}\right) / \log\left(p_{\text{SMT}}^{\ell^\star} p_{\text{SD}}^{\ell^\star} p_{\text{Q}}^{\ell^\star}\right)$ where $\ell^\star$ is the locus ℓ that achieves the largest combined LOCATER p-value. The proportion of signal attributed to SD (dashed lines) or quadratic form testing, abbreviated as Q (dotted lines), is defined analogously. Proportions are displayed as a function of total association signal strength s: the $-\log_{10}$ p-value that one would obtain by testing with an oracle ANOVA model that "knows" the causal variants.

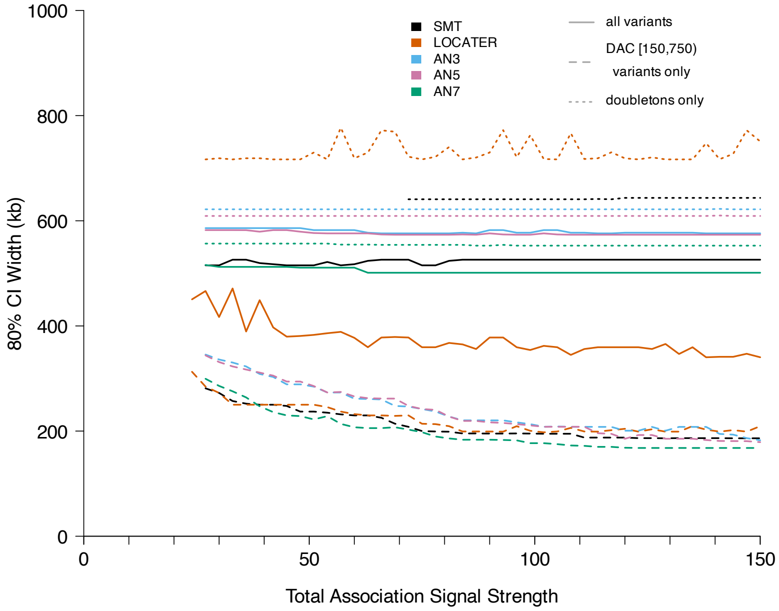

Figure 13: Width of confidence intervals (CI), centered on the position with the largest signal, that cover the midpoint of the causal region in 80% of simulations where there are 3 causal variants, all hidden. In simulations where multiple variants are tied to have the largest association signal, we take the distance to the midpoint of the causal region to be the average distance from each of the tied variants. CI width is displayed as a function of total association signal strength s: the $-\log_{10}$ p-value that one would obtain by testing with an oracle ANOVA model that "knows" the causal variants.

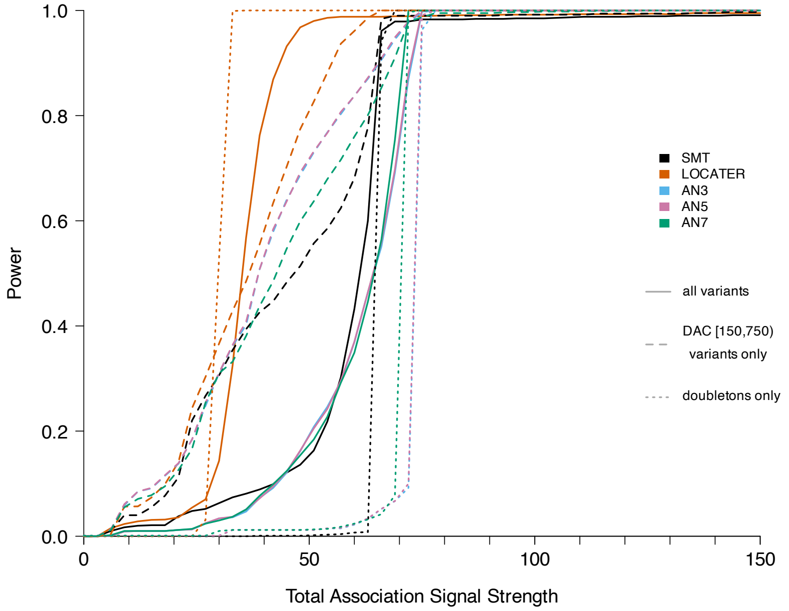

Figure 14: Power comparison between SMT, ARG-Needle, and LOCATER in simulations where there are 9 causal variants, all observed. Power is displayed as a function of total association signal strength s: the $-\log_{10}$ p-value that one would obtain by testing with an oracle ANOVA model that "knows" the causal variants.

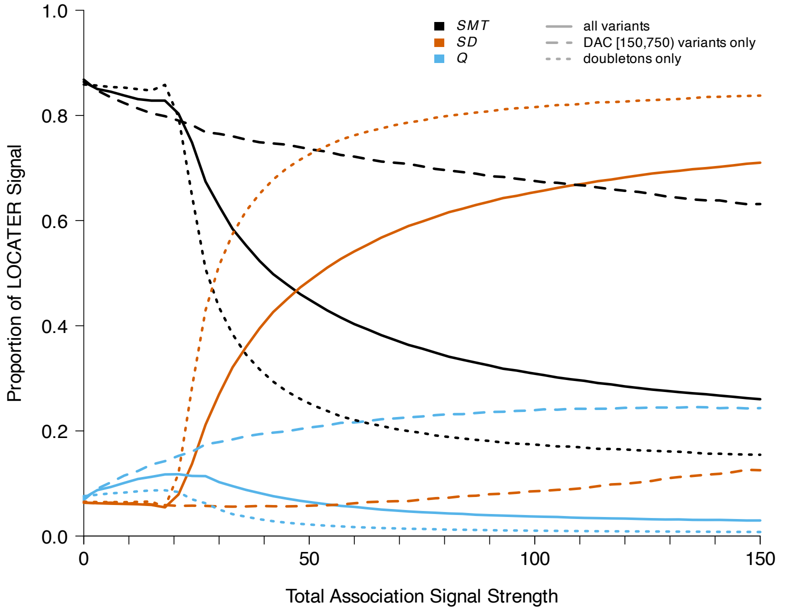

Figure 15: Average proportion of signal driven by each sub-test within LOCATER in simulations where there are 9 causal variants, all observed. For every simulation, the proportion of signal attributed to the SMT part of LOCATER (solid lines) defined as $\log\left(p_{\mathrm{SMT}}^{\ell^\star}\right) / \log\left(p_{\mathrm{SMT}}^{\ell^\star} p_{\mathrm{SD}}^{\ell^\star} p_{\mathrm{Q}}^{\ell^\star}\right)$ where $\ell^\star$ is the locus ℓ that achieves the largest combined LOCATER p-value. The proportion of signal attributed to SD (dashed lines) or quadratic form testing, abbreviated as Q (dotted lines), is defined analogously. Proportions are displayed as a function of total association signal strength s: the $-\log_{10}$ p-value that one would obtain by testing with an oracle ANOVA model that "knows" the causal variants.

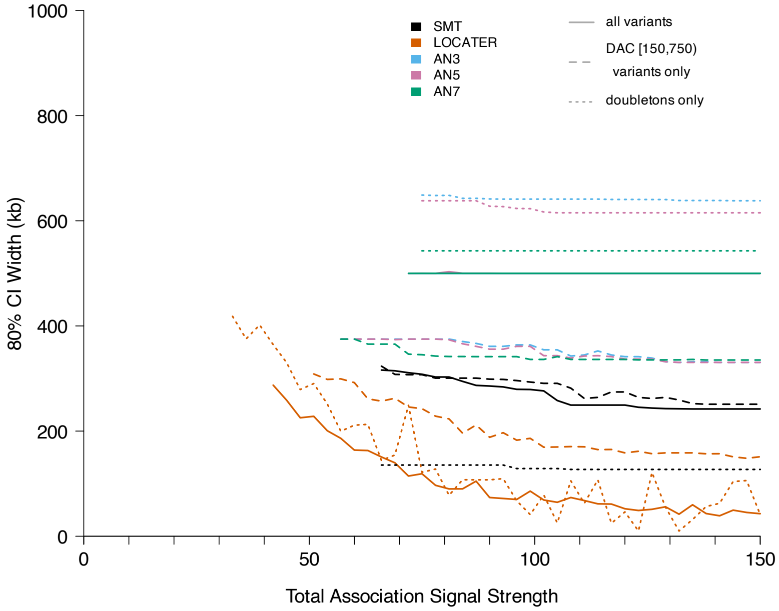

Figure 16: Width of confidence intervals (CI), centered on the position with the largest signal, that cover the midpoint of the causal region in 80% of simulations where there are 9 causal variants, all observed. In simulations where multiple variants are tied to have the largest association signal, we take the distance to the midpoint of the causal region to be the average distance from each of the tied variants. CI width is displayed as a function of total association signal strength s: the $-\log_{10}$ p-value that one would obtain by testing with an oracle ANOVA model that "knows" the causal variants.

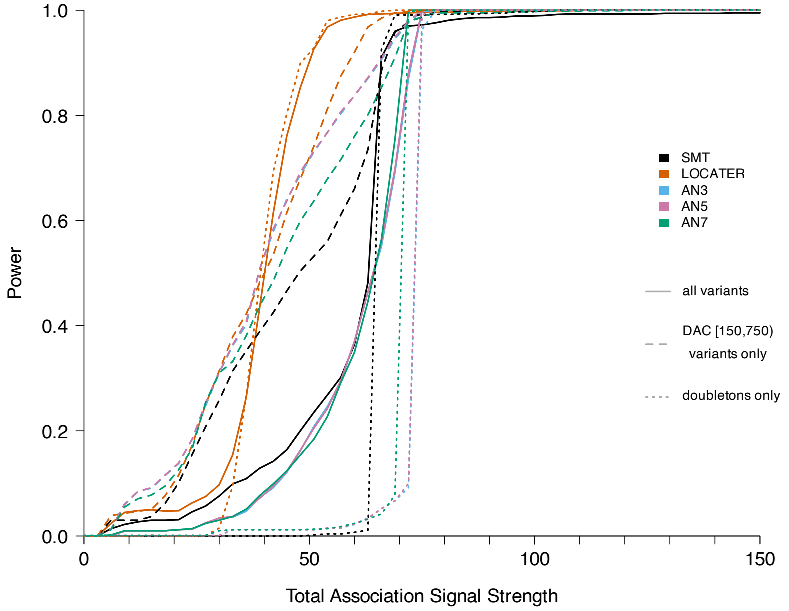

Figure 17: Power comparison between SMT, ARG-Needle, and LOCATER in simulations where there are 9 causal variants, all hidden. Power is displayed as a function of total association signal strength s: the $-\log_{10}$ p-value that one would obtain by testing with an oracle ANOVA model that "knows" the causal variants.

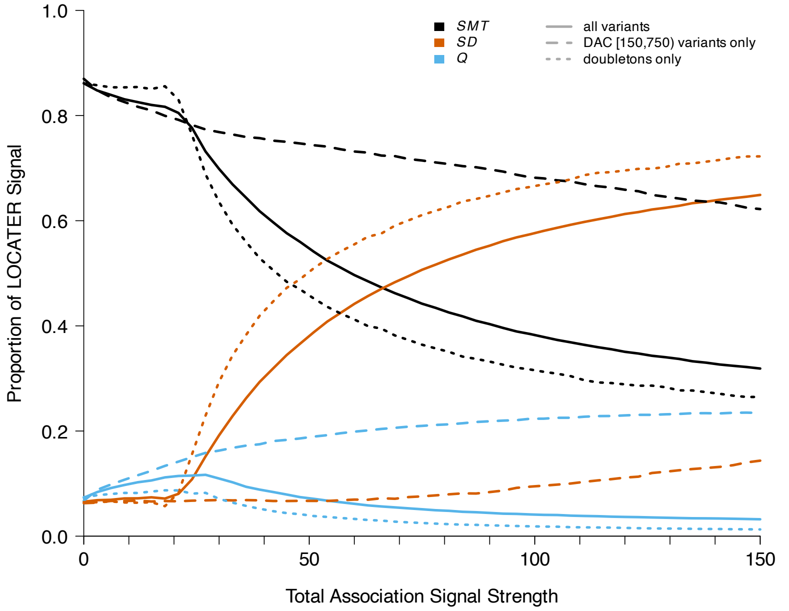

Figure 18: Average proportion of signal driven by each sub-test within LOCATER in simulations where there are 9 causal variants, all hidden. For every simulation, the proportion of signal attributed to the SMT part of LOCATER (solid lines) defined as $\log\left(p_{\mathrm{SMT}}^{\ell^\star}\right) / \log\left(p_{\mathrm{SMT}}^{\ell^\star} p_{\mathrm{SD}}^{\ell^\star} p_{\mathrm{Q}}^{\ell^\star}\right)$ where $\ell^\star$ is the locus ℓ that achieves the largest combined LOCATER p-value. The proportion of signal attributed to SD (dashed lines) or quadratic form testing, abbreviated as Q (dotted lines), is defined analogously. Proportions are displayed as a function of total association signal strength s: the $-\log_{10}$ p-value that one would obtain by testing with an oracle ANOVA model that "knows" the causal variants.

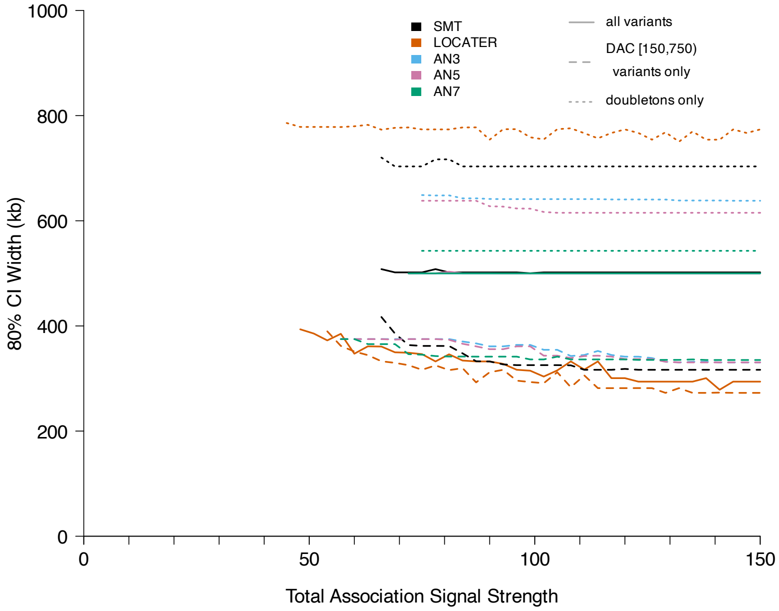

Figure 19: Width of confidence intervals (CI), centered on the position with the largest signal, that cover the midpoint of the causal region in 80% of simulations where there are 9 causal variants, all hidden. In simulations where multiple variants are tied to have the largest association signal, we take the distance to the midpoint of the causal region to be the average distance from each of the tied variants. CI width is displayed as a function of total association signal strength s: the $-\log_{10}$ p-value that one would obtain by testing with an oracle ANOVA model that "knows" the causal variants.

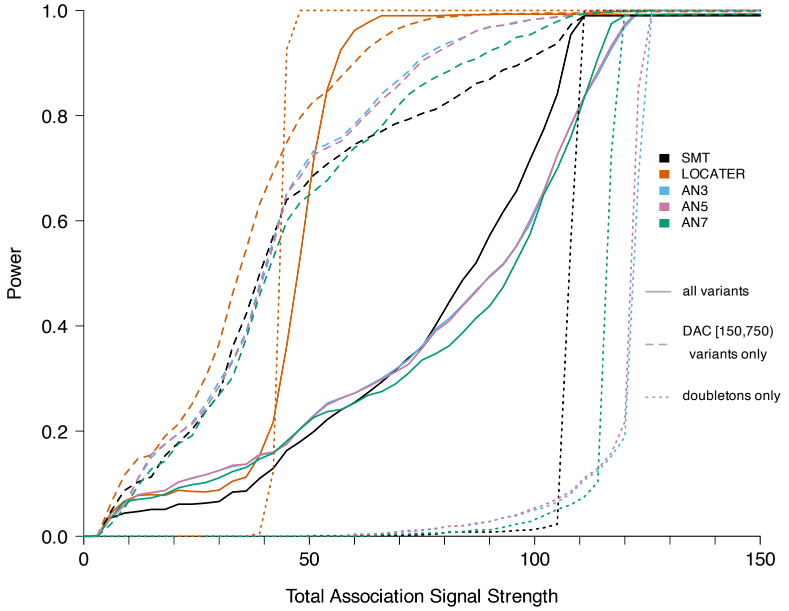

Figure 20: Power comparison between SMT, ARG-Needle, and LOCATER in simulations where there are 15 causal variants, all observed. Power is displayed as a function of total association signal strength s: the $-\log_{10}$ p-value that one would obtain by testing with an oracle ANOVA model that "knows" the causal variants.

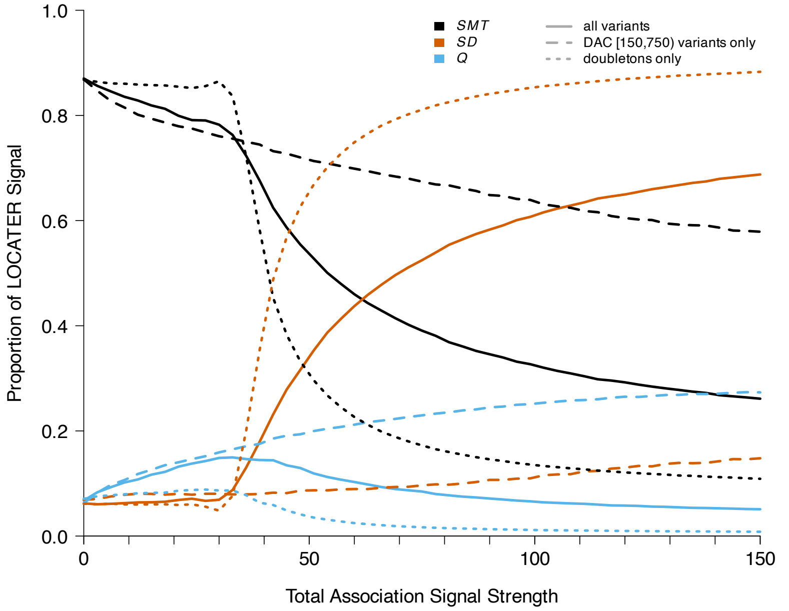

Figure 21: Average proportion of signal driven by each sub-test within LOCATER in simulations where there are 15 causal variants, all observed. For every simulation, the proportion of signal attributed to the SMT part of LOCATER (solid lines) defined as $\log\left(p_{\text{SMT}}^{\ell^\star}\right) / \log\left(p_{\text{SMT}}^{\ell^\star} p_{\text{SD}}^{\ell^\star} p_{\text{Q}}^{\ell^\star}\right)$ where $\ell^\star$ is the locus ℓ that achieves the largest combined LOCATER p-value. The proportion of signal attributed to SD (dashed lines) or quadratic form testing, abbreviated as Q (dotted lines), is defined analogously. Proportions are displayed as a function of total association signal strength s: the $-\log_{10}$ p-value that one would obtain by testing with an oracle ANOVA model that "knows" the causal variants.

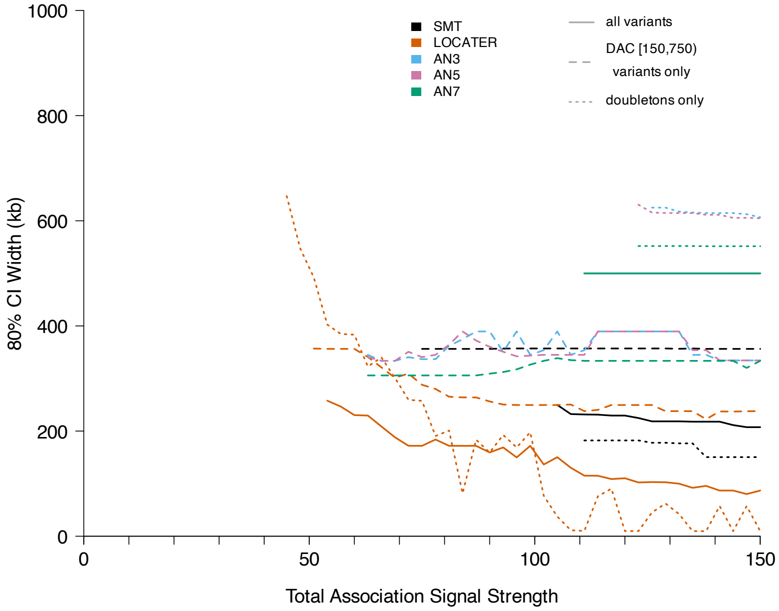

Figure 22: Width of confidence intervals (CI), centered on the position with the largest signal, that cover the midpoint of the causal region in 80% of simulations where there are 15 causal variants, all observed. In simulations where multiple variants are tied to have the largest association signal, we take the distance to the midpoint of the causal region to be the average distance from each of the tied variants. CI width is displayed as a function of total association signal strength s: the $-\log_{10}$ p-value that one would obtain by testing with an oracle ANOVA model that "knows" the causal variants.

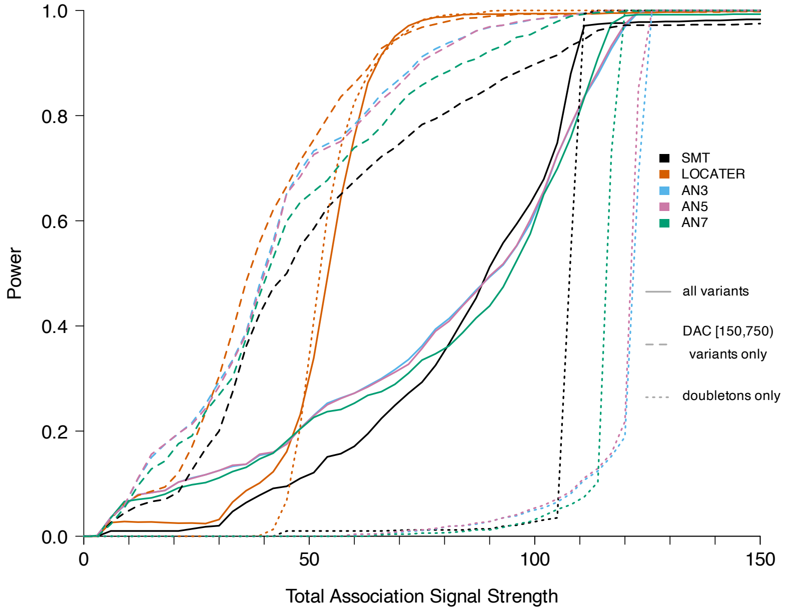

Figure 23: Power comparison between SMT, ARG-Needle, and LOCATER in simulations where there are 15 causal variants, all hidden. Power is displayed as a function of total association signal strength s: the $-\log_{10}$ p-value that one would obtain by testing with an oracle ANOVA model that "knows" the causal variants.

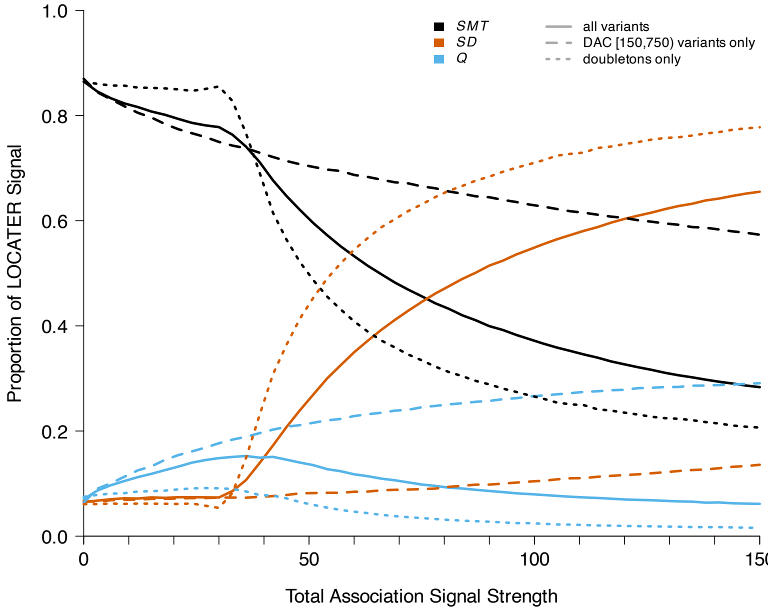

Figure 24: Average proportion of signal driven by each sub-test within LOCATER in simulations where there are 15 causal variants, all hidden. For every simulation, the proportion of signal attributed to the SMT part of LOCATER (solid lines) defined as $\log\left(p_{\mathrm{SMT}}^{\ell^\star}\right) / \log\left(p_{\mathrm{SMT}}^{\ell^\star} p_{\mathrm{SD}}^{\ell^\star} p_{\mathrm{Q}}^{\ell^\star}\right)$ where $\ell^\star$ is the locus ℓ that achieves the largest combined LOCATER p-value. The proportion of signal attributed to SD (dashed lines) or quadratic form testing, abbreviated as Q (dotted lines), is defined analogously. Proportions are displayed as a function of total association signal strength s: the $-\log_{10}$ p-value that one would obtain by testing with an oracle ANOVA model that "knows" the causal variants.

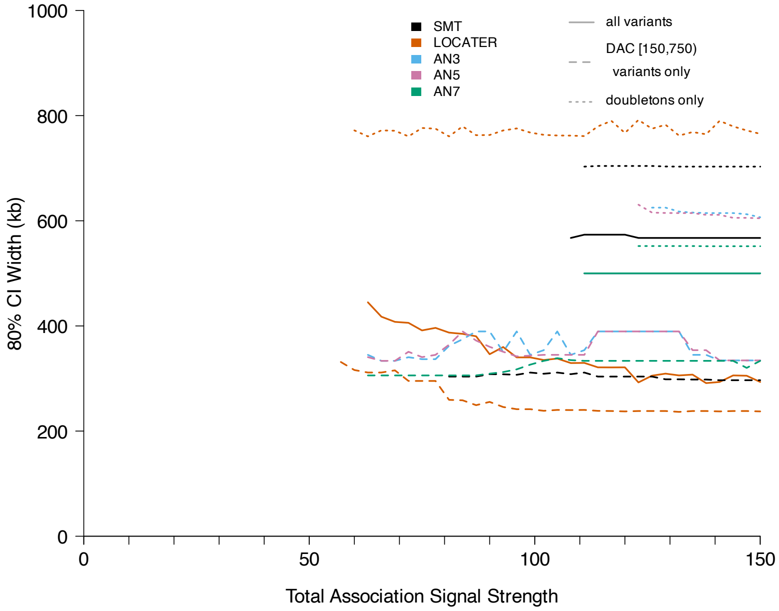

Figure 25: Width of confidence intervals (CI), centered on the position with the largest signal, that cover the midpoint of the causal region in 80% of simulations where there are 15 causal variants, all hidden. In simulations where multiple variants are tied to have the largest association signal, we take the distance to the midpoint of the causal region to be the average distance from each of the tied variants. CI width is displayed as a function of total association signal strength s: the $-\log_{10}$ p-value that one would obtain by testing with an oracle ANOVA model that "knows" the causal variants.

1.5 Power Curves: 100 kb Causal Window

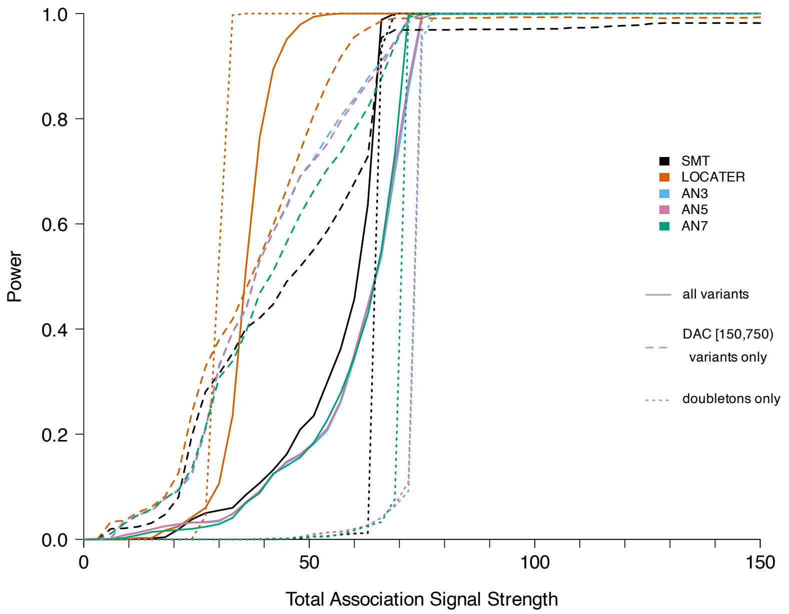

Figure 26: Power comparison between SMT and LOCATER in simulations where there are 9 causal variants within a 100 kb causal window, all observed. Compare to Figure 14. Power is displayed as a function of total association signal strength s: the $-\log_{10}$ p-value that one would obtain by testing with an oracle ANOVA model that "knows" the causal variants.

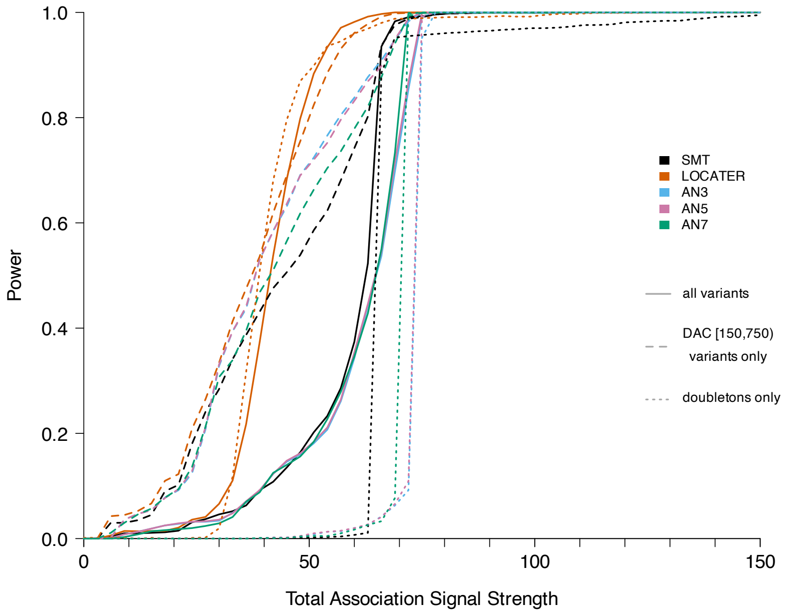

Figure 27: Power comparison between SMT and LOCATER in simulations where there are 9 causal variants within a 100 kb causal window, all hidden. Compare to Figure 17. Power is displayed as a function of total association signal strength s: the $-\log_{10}$ p-value that one would obtain by testing with an oracle ANOVA model that "knows" the causal variants.

2 Supplementary Methods

2.1 Running kalis

As described in [2, 3, 4], the LS model requires a pre-defined recombination map and two model parameters: μ governing the probability of mutations and N_e recombination events. Throughout this paper, we provided `kalis` with the true recombination map under which haplotypes were simulated. Throughout we set $\mu = 10^{-4}$ and $N_e = 10^{-16}$.

2.2 Connecting our Generalized eGRM to the standard eGRM

Here we explain the connection between our generalized eGRM matrix to the standard eGRM, as presented in Equation 1 in [5]. In the Defining our Generalized Relatedness Matrix subsection of the Methods in the main text, we define a local haplotype relatedness matrix $\Psi^{(\ell)} \in \mathbb{R}_{\geq 0}^{N \times N}$ with elements

$$\Psi_{ij}^{(\ell)} = \sum_{k=\pi_j^{-1}(i)}^{N} \psi_j(k) \quad \text{where} \quad \psi_j(k) = \frac{1}{k} g_k \left(d_{(k+1)j}^{(\ell)} - d_{kj}^{(\ell)} \right) \tag{10}$$

and our generalized eGRM in terms of Equation (10) as

$$\Omega^{(\ell)} = \text{sym} \left(B_{n,2}^{\top} \Psi^{(\ell)} B_{n,2} \right) \tag{11}$$

where $\text{sym}(M) = \frac{1}{2}\left(M + M^{\top}\right)$ is the symmetric part of a square matrix M, $B_{n,2} = I_{n \times n} \otimes \mathbf{1}_2$, and $\otimes$ is the Kronecker product. For clarity, throughout this section we will assume that we are considering a particular target locus ℓ and suppress indexing objects by ℓ.

Let n be the number of samples in a genetic dataset. Let

$$\mathcal{H}_i = \{ j \in \mathbb{N} : \text{haplotype } j \text{ belongs to sample } i \} \tag{12}$$

and $\Delta_i = |\mathcal{H}_i|$ be the ploidy of sample i so that $N = \sum_i \Delta_i$ is the total number of haplotypes in the sample. Let $H \in \{0,1\}^{N \times p}$ be the haplotype matrix encoding the carriers of p in a genomic region centered on locus ℓ or on an inferred local ancestral tree at ℓ as in [5]. Let $f_k = \sum_{i=1}^{n} H_{ik}$ be the allele frequency of the kth variant. Given a set of weights $w \in \mathbb{R}_{\geq 0}^{p}$ which may be based on inferred clade probabilities or otherwise, we start with a weighted haplotype relatedness matrix

$$\Psi' = \sum_{k=1}^{p} \frac{w_k}{f_k} H_{\cdot j} H_{\cdot k}^{\top}. \tag{13}$$

We can write this more compactly as $\Psi' = \widetilde{H}\widetilde{H}^{\top}$ where $\widetilde{H} = H \text{diag}\left(\sqrt{w_k/f_k}\right)$. Assuming an additive model, we can collapse this weighted haplotype relatedness matrix to a weighted genotype relatedness matrix

$$\Omega'_{ij} = \sum_{k \in \mathcal{H}_i} \sum_{l \in \mathcal{H}_j} \Psi'_{kl}. \tag{14}$$

For simplicity, from here forward we will assume that (1) the ploidy of each sample Δ_i is the same constant ploidy Δ for all samples i and (2) the rows and columns of Ψ' are permuted such that haplotypes from the same sample are grouped together. This allows us to more succinctly write $\Omega' = B_{n,\Delta}^{\top} \Psi' B_{n,\Delta}$ where $B_{n,\Delta} = I_{n \times n} \otimes \mathbf{1}_{\Delta}$ and $\otimes$ is the Kronecker product. This gives us

$$\begin{aligned}
\Omega' &= B_{n,\Delta}^{\top} \left(\sum_{k=1}^{p} \frac{w_k}{f_k} H_{\cdot k} H_{\cdot k}^{\top} \right) B_{n,\Delta} \\
&= \sum_{k=1}^{p} \frac{w_k}{f_k} B_{n,\Delta}^{\top} H_{\cdot k} H_{\cdot k}^{\top} B_{n,\Delta} \\
&= \sum_{k=1}^{p} \frac{w_k}{f_k} G_{\cdot k} G_{\cdot k}^{\top} \\
&= G \text{diag}\left(w_k/f_k\right) G^{\top}
\end{aligned}$$

where $G\{0,1,2\}^{n\times p}$ is the genotype matrix corresponding to the p variants on the local tree. Now if we consider a model without any background covariates besides an intercept, we have $P = I - \frac{1}{n}\mathbf{1}\mathbf{1}^\top$.

$$\begin{aligned} P\Omega' P &= PG\text{diag}\left(w_k/f_k\right)G^\top P \\ &= \sum_{k=1}^{p} \frac{w_k}{f_k} PG_{\cdot k}\left(PG_{\cdot k}\right)^\top \end{aligned} \tag{15}$$

The standard GRM assumes that each variant is in Hardy–Weinberg equilibrium. So in order to compare Equation (15) to the standard GRM, let us also assume that each of our p variants is in perfect Hardy–Weinberg equilibrium. That means each centered genotype vector $PG_{\cdot k} = G_{\cdot k} - 2f_k\mathbf{1}$, allowing us to write

$$\begin{aligned} \left(P\Omega' P\right)_{ij}\big|_{HW} &= \sum_{k=1}^{p} w_k \frac{\left(G_{ik}-2f_k\right)\left(G_{jk}-2f_k\right)}{f_k} \\ &= \frac{1}{p}\sum_{k=1}^{p} w_k \eta\left(f_k\right)\frac{\left(G_{ik}-2f_k\right)\left(G_{jk}-2f_k\right)}{2f_k\left(1-f_k\right)} \end{aligned} \tag{16}$$

where $\eta\left(f_k\right) = 2p\left(1-f_k\right)$. The standard GRM defined in Equation 1 of [5] is just a simple special case of Equation (16) where $\eta\left(f_k\right) = 1$ for all arguments f_k and $w_k = 1$ for all variants. The eGRM allows for non-equal weights w_k. Some simple algebra shows that our choice of a $\frac{1}{k}$ in our definition of ψ in Equation 10 corresponds to $\eta\left(f_k\right) = 2p\left(1-f_k\right)$. We chose this weighting in order to upweight variants that have low derived allele frequency. Under mild selection pressure, we expect very common derived alleles to have smaller phenotypic effects. With very minimal modification of our current implementation, any weighting function of the derived allele frequency η may be used. We plan to make such a general weighting function available in future versions of LOCATER.

Having rewritten the eGRM in terms of Ψ', let us connect Ψ' to Ψ from Equation (10), which we use in our generalized eGRM construction. Consider the special case where we have a chromosome without recombination so that our genetic distance matrix $d^{(\ell)}$ reflects a single marginal tree, making all clade calls are aligned. If we also set each g_k to the identity function and assume consistency across columns of our distance matrix so that $d^{(\ell)}_{(k+1)j} - d^{(\ell)}_{kj} = w_k$ for all haplotypes j carrying a variant of frequency k, then Ψ as defined in Equation (10) would be symmetric and proportional to the the weighted haplotype matrix Ψ' as defined in Equation (13). As a consequence, the similarity matrix we would obtain $B_{n,d}^\top \Psi B_{n,d}$ would equal $\Omega' = B_{n,d}^\top \Psi' B_{n,d}$. However, due to recombination, the distance matrix Ψ is not symmetric and the distances across columns may not be consistent with a single tree structure. Rather than attempting to synchronize the distances by clustering them into a tree structure, we trust the LS model, taking the distances as representing a weighted convolution of nearby tree structures.

2.3 Optimal Checkpointing Scheduler

Let j index the positions of our L target loci from smallest to largest. We will solve for an optimal checkpointing schedule for the forward algorithm to propagate to target loci sequentially in reverse order, from $j = L$ to $j = 1$. We will use $j = 0$ to index the HMM prior hidden state probabilities used to initialize the forward algorithm. Informally, for each subsequent target locus, a checkpointing schedule states the checkpoint at which the forward algorithm should start and the intervening target loci where it should stop to store any new checkpoints on its way to reach that target locus. To optimize this schedule, we do not need to consider storing checkpoints at variants that are not target loci since any such checkpointing strategy could be improved by moving those checkpoints to target loci. Thus, our checkpointing schedule will only need to render integers in $1,\ldots,L$.

Optimizing the checkpointing schedule is based on recycling the memory used to store checkpoints that are no longer being used. We solve for an optimal checkpointing schedule where the computational cost to propagate the forward algorithm from any position to any other may be arbitrary. The checkpointing routine that minimizes the total computational cost in this setting may be obtained by solving nested optimization problems. While tractable, solving this system of nested optimization problems can be rather time consuming

to solve. Therefore, we subsequently consider the special case where the cost of propagating the forward algorithm between consecutive target positions is constant. For example, in a genomics context this may be true if we have equally spaced target loci. In that special case, we explain how the optimal checkpointing schedule can be solved rapidly via dynamic programming.

We start with the general case. Define $g : \mathbb{Z}^2_{\geq 0} \to \mathbb{R}_{\geq 0}$ such that $g(s,t)$ is the cost of propagating the forward algorithm directly from target locus s to target locus t, defined to be zero whenever $s = t$. Define $f : \mathbb{Z}^3_{\geq 0} \to \mathbb{R}_{\geq 0}$ such that $f(s,t,c)$ is the minimal cost required for the forward algorithm to sequentially visit target loci t, $t-1$, $t-2, \dots, s$ given c available checkpoints and a known set of forward probabilities (i.e. a checkpoint, or the HMM prior) at s. That is, $f(s,t,c)$ corresponds to the cost of the (unknown) optimal schedule given memory for c checkpoints.

We aim to obtain a checkpoint schedule which enables us to achieve the cost $f(0,T,C)$. This objective is defined by the three following equations. First, if there are no checkpoints available, simply propagating to each target locus sequentially from the initial position is our only option, which comes with cost $f(x,y,0) = \sum_{j=x}^{y} g(x,j)$. Second, if we already know the forward probabilities at a given position, there is no computational cost to obtaining them, so $f(x,x,c) = 0$ for any c. Third, we have the following recursive relationship

$$f(s,t,c) = \min_{j \in \mathbb{Z}_{\geq 0} : j \in (s,t]} g(s,j) + f(s,j-1,c) + f(j,t,c-1)\,. \tag{17}$$

Intuitively, this recursion expresses the idea that solving for an optimal checkpointing schedule with c checkpoints over an interval can be thought of as placing one of the checkpoints at some optimal index to divide the interval so that the upper part is solved with $c-1$ checkpoints; the lower part, c checkpoints. We will write that optimal index, the argument that achieves the minimum of Equation (17) as

$$h(s,t,c) = \operatorname*{argmin}_{j \in \mathbb{Z}_{\geq 0} : j \in (s,t]} g(s,j) + f(s,j-1,c) + f(j,t,c-1)\,. \tag{18}$$

Together, these three equations allow us to solve for an optimal checkpoint schedule by transversing a bifurcating tree from right to left, from the leaves toward the root, where each node can be identified with an optimal index h.

While solving this recursion is tractable, it can be rapidly accelerated if we assume that the computational cost of propagating the forward probabilities between adjacent target loci is fixed. In other words, we assume $g(x-1,x)$ is constant for all target indices $x \in \{1,2,\dots,L\}$. This makes the computational cost a simple linear function of the distance between indices, yielding the following simplified system of equations.

Analogous to above, we have $\widetilde{f}(i,0) = i(i+1)/2$ and $\widetilde{f}(0,c) = 0$ for all c. Our recursions simplify to

$$\widetilde{f}(i,c) = \min_{j \in \mathbb{Z}_{\geq 0} : j \leq i} j + \widetilde{f}(j-1,c) + \widetilde{f}(i-j,c-1)\,, \tag{19}$$

$$\widetilde{h}(i,c) = \operatorname*{argmin}_{j \in \mathbb{Z}_{\geq 0} : j \leq i} j + \widetilde{f}(j-1,c) + \widetilde{f}(i-j,c-1)\,. \tag{20}$$

For some pre-specified maximum number of target loci L and maximum number of available checkpoints C, let us define two matrices that we will use as look-up tables for our dynamic program. Let $F \in \mathbb{R}^{(L+1)\times(C+1)}_{\geq 0}$ such that $F_{ij} = \widetilde{f}(i-1,j-1)$ and $H \in \mathbb{Z}^{(L+1)\times(C+1)}_{\geq 0}$ such that $H_{ij} = \widetilde{h}(i-1,j-1)$. Given the nested nature of 19 and 20, we can rapidly solve for each entry of F and H by synchronously iterating over the entries of both matrices in column-major order. Once we have a completed F and H, we can use 20 to read off the appropriate entries in table H to obtain the optimal checkpointing schedule for any problem with up to L target variants and C available checkpoints. Methods for constructing H and reading it to construct a checkpoint schedule are available in `kalis 2.0`. This implementation covers the slightly more general case than (19) where the cost depends on distance but is independent of locus. This corresponds to a translation invariance assumption that does not cover the most general case in (17).

2.4 Robust Tail Approximation

The rate of tail decay under Satterthwaite approximation, $(2\alpha)^{-1}$, tends to chronically over-estimate the true rate of decay in the tails of R_k, which is $\left(2\max_{j>k}\lambda_j\right)^{-1}$. In fact, in the positive semi-definite case, the Hölder Inequality (with $p=1, q=\infty$) shows that

$$(2\alpha)^{-1} = \left(\frac{2\sum_{j>k}\lambda_j^2}{\sum_{j>k}\lambda_j}\right)^{-1} = \left(\frac{2\sum_{j>k}|\lambda_j\lambda_j|}{\sum_{j>k}|\lambda_j|}\right)^{-1} \geq \left(\frac{2\left(\sum_{j>k}|\lambda_j|\right)\max_{j>k}|\lambda_j|}{\sum_{j>k}|\lambda_j|}\right)^{-1} = \left(2\max_{j>k}\lambda_j\right)^{-1}. \tag{21}$$

Our proposed approximation $\ddot{R}_k$ addresses this problem by using tails that decay at least as slowly as R_k. However, this alone does not guarantee accurate p-value estimation across the range of quantiles required for LOCATER. Our goal is to estimate $-\log$ p-values given by $-\log\left(1-F_{R_k}(x)\right)$ where F_{R_k} is the CDF of R_k. Since R_k has exponential tails, $-\log\left(1-F_{R_k}(x)\right)$ is a linear function of x for large x, with a slope determined the rate of decay in the tail and an intercept that is determined by the body of the distribution of R_k. Thus, estimating the body of the distribution of R_k is required for accurately estimating p-values in the tail of the distribution of R_k.

We will make use of the observation that the spectrum of a typical $P\Omega^{(\ell)}P$ observed in our genomics application has the following three-part structure. First the spectrum decays over the leading ten or so eigenvalues. This segment of the spectrum corresponds to large-scale population structure, orthogonal to the columns of A, encoded by $\Omega^{(\ell)}$. Then the spectrum levels off to a plateau where the eigenvalues are all roughly of the same magnitude. This second segment of the spectrum corresponds to the fine-scale population structure encoded by $\Omega^{(\ell)}$. Most eigenvalues in this second segment of the spectrum are positive with occasional negative eigenvalues interspersed. Eventually this plateau in the spectrum gives way to the third and final segment of the spectrum where the magnitude of the eigenvalues decays to zero.

Our strategy for estimating tail probabilities for $Y^\top P\Omega^{(\ell)}PY$ is to explicitly evaluate the eigenvalues in the first segment of the spectrum, placing them into T_k, so that we can treat R_k as the sum of terms arising from the second segment and third segment.

In regions with high local recombination rates, within hotspots, there are relatively few proximal mutations that are informative about the local genealogy. Thus, the spectrum of $P\Omega^{(\ell)}P$ decays rapidly in these regions. In these cases we can easily evaluate enough eigenvalues so that R_k explains little of the variance of T_k+R_k. At these loci, approximating R_k with $\ddot{R}_k$ is more than sufficient to calculate p-values for $T_k+R_k = Y^\top P\Omega^{(\ell)}PY$. Almost any unimodal distribution matching the first and second moments would work for approximating R_k in this high-recombination context, which is achieved by our moment matching of $\ddot{R}_k$.

However, at the vast majority of loci along the genome are in between recombination hotspots. There the spectrum of a typical $P\Omega^{(\ell)}P$ tends to decay over the leading ten or so eigenvalues and then level off to a very long plateau of eigenvalues, the vast majority of which are positive. This plateau arises from the abundance of rare variants encoding small clades in $\Omega^{(\ell)}$. While eventually this plateau decays to zero, the eigenvalues within the plateau segment of the spectrum make up the vast majority of the total sum of squared eigenvalues. As a consequence, the contribution of R_k to the overall variance of T_k+R_k is much larger than the contribution of T_k for any tractable k. Thus, to estimate accurate tail probabilities for T_k+R_k, it is imperative that we do not underestimate the tail probabilities of R_k.

To do this, we must ensure that we have control of the body of the distribution. When we top-k eigendecompose $P\Omega^{(\ell)}P$ in LOCATER, we increase the number of eigenvalues k we evaluate until we reach the following stopping criteria.

$$k^\star = \inf\left\{k \in [n-q] : \left(\sum_{j=1}^{k}\lambda_j^2 \Big/ \sum_{j=1}^{n-q}\lambda_j^2\right) \geq 0.95 \text{ or } \left(\lambda_k/\lambda_{k-1}\right)^2 \geq 0.95\right\}. \tag{22}$$

In other words, when we want precise p-values, we compute the top $k \geq k^\star$ eigenvalues of PMP so that

$$T_k = \sum_{j=1}^{k^\star}\lambda_j Z_j^2 + \sum_{j=k^\star+1}^{k}\lambda_j Z_j^2. \tag{23}$$

The first term in our stopping criteria catches the high-recombination case where R_k makes only a small contribution to the distribution of $T_k + R_k$. The second term in our stopping criteria catches the more typical case where the spectrum decays to a long plateau and ensures that we eigendecompose until that plateau is reached. Once the plateau is reached, all of leading terms with relatively large eigenvalues have been moved into T_k. This leaves R_k consisting of many terms with nearly equal eigenvalues. Since these terms have nearly equal variance, by the Berry–Esseen Theorem [6, 7], the body of R_k will be very close to a Gaussian. Since the Gaussian distribution is determined by its the mean and variance, this means that we can reliably estimate the body of the distribution with an approximate distribution that is also Gaussian in the body (with the appropriate mean and variance). Given the preponderance of positive eigenvalues in the plateau, minimizing $|\mu|$ when selecting (a, b, μ) (Section 2.5), ensures a large value of a for $\ddot{R}_k$. This guarantees that the body of the distribution of $\ddot{R}_k$ will also be very close to Gaussian. Thus $\ddot{R}_k$ reliably matches the body of the target null distribution. In combination with the robust rate of decay in the right tail $\ddot{R}_k$, this ensures reliable p-value estimation even for very small tail probabilities.

2.5 Accounting for Inflation Driven by Unmeasured Confounders & Polygenicity

Consider the presence of some unobserved variable $B \in \mathbb{R}^n$. Without loss of generality, assume $\|B\|_2 = \sqrt{n-q}$ and $PB = B$. For some scalar γ, we have $Y = A\alpha + \gamma B + \sigma\epsilon$. Then our estimator $\hat{\sigma}^2 = Y^\top PY/(n-q) \xrightarrow{P} \gamma^2 + \sigma^2$. This gives us the null distribution

$$\begin{aligned}
\left(\gamma^2+\sigma^2\right)^{-1} Y^\top PMPY &= \left(\gamma^2+\sigma^2\right)^{-1} \sum_{j=1}^{n-q} \lambda_j \left(\sigma V_j^\top P\epsilon + \gamma V_j^\top B\right)^2 \\
&\sim \frac{\sigma^2}{\gamma^2+\sigma^2} \sum_{j=1}^{n-q} \lambda_j \left(Z_j + \frac{\gamma}{\sigma} V_j^\top B\right)^2 \\
&= \nu \sum_{j=1}^{n-q} \lambda_j \left(Z_j + \delta_j\right)^2 \\
&\sim \nu \sum_{j=1}^{n-q} \lambda_j W_j
\end{aligned}$$

where $\nu = \frac{\sigma^2}{\gamma^2+\sigma^2}$, $\delta_j = \frac{\gamma}{\sigma} V_j^\top B$, and each $W_j \sim \chi_1^2\left(\delta_j^2\right)$, independent. The parameter $\nu \in [0, 1]$ captures the proportion of the variance of Y that is not explained by the unobserved variable B and each $\left(\delta_j^2\right)_{j=1}^{n-q}$ captures how correlated the clade structure encoded in $\Omega^{(\ell)}$ is with the unobserved variable B.

We can gather information about ν by looking at the distribution of this quadratic form test statistic at a grid of loci spaced far apart along the genome. Unfortunately, we cannot attempt to estimate each individual δ_j in a similar way since each specific δ_j depends on the precise j^{th} eigenvector V_j calculated at a given position. However, the spherical symmetry of the multivariate normal distribution helps us tackle this problem.

If all of the eigenvalues, λ_j were equal, our null distribution would have a scaled non-central chi-square distribution with $n - q$ degrees of freedom and a single non-centrality parameter $\sum_{j=1}^{n-q} \delta_j^2$. This collapse into a single non-centrality parameter means that we would not need to model each individual δ_j^2, but rather just the average δ_j^2.

To interpret this average, again, assuming all of the eigenvalues were equal,

$$\sum_{j=1}^{n-q} \delta_j^2 = \frac{\gamma^2}{\sigma^2} \sum_{j=1}^{n-q} \left(V_j^\top B\right)^2 \propto B^\top PMPB = B^\top MB. \tag{24}$$

Thus $\sum_{j=1}^{n-q} \delta_j^2$ can be thought as proportional to the quadratic form test statistic we would obtain taking B as our phenotype vector, which is a measure of how correlated B is with the structure encoded in $\Omega^{(\ell)}$. This suggests a second, related interpretation. Recall B and all V_js are scaled and that the inclusion of an intercept

in A ensures that B and each V_j are centered. This means that $\sum_{j=1}^{n-q} \delta_j^2$ is proportional to average correlation between B and each V_j.

$$\sum_{j=1}^{n-q} \delta_j^2 = \frac{\gamma^2}{\sigma^2} \sum_{j=1}^{n-q} \left(V_j^\top B\right)^2 = \frac{n\gamma^2}{\sigma^2} \frac{1}{n} \sum_{j=1}^{n-q} \mathrm{Corr}\left(V_j, B\right)^2 = \frac{n\gamma^2}{\sigma^2} \overline{\mathrm{Corr}\left(V_j, B\right)^2}. \tag{25}$$

This is proportional to the coefficient of multiple correlation (R^2) one would obtain by regressing B onto the eigenvectors V under the ordinary least squares model.

Since we expect that the typical correlation between B and each V_j may be different for V_js capturing common variant structure versus rare variant structure, we replace each δ_j^2 such that

$$W_j \sim \begin{cases} \chi_1^2\left(\delta_\star^2\right) & j \leq k^\star \\ \chi_1^2\left(\delta_\dagger^2\right) & j > k^\star. \end{cases}$$

This leaves us with a null distribution with three scalar inflation parameters, $\nu, \delta_\star^2, \delta_\dagger^2 \in \mathbb{R}_{\geq 0}$. In summary, we have the following interpretations.

- ν is the proportion of variance in Y *not* explained by unobserved confounders
- $\gamma_\star^2$ is proportional to the average correlation between unobserved confounders and the large-scale haplotype structure encoded in $\Omega^{(\ell)}$ (that is not included in A)
- $\gamma_\dagger^2$ is proportional to the average correlation between unobserved confounders and the fine scale haplotype structure encoded in $\Omega^{(\ell)}$

Now we turn to estimating these parameters. We can use $\mathrm{tr}\left(PMP\right)$ and $\|PMP\|_{HS}^2$ to calculate the mean and variance of R_k. Using those moments, we approximate R_k with a shifted difference of chi-square random variables. Let

$$\ddot{R}_k = \nu \left|\lambda_k\right| \left(X_{a,\delta_\dagger^2} - X'_{b,\delta_\dagger^2} + \mu\left(1+\delta_\dagger^2\right)\right)$$

where $X_{a,\delta_\dagger^2} \sim \chi_a^2\left(a\delta_\dagger^2\right)$ is distributed chi-squared with a degrees of freedom and non-centrality parameter $a\delta_\dagger^2$ and independent of $X'_{b,\delta^2\dagger} \sim \chi_b^2\left(b\delta_\dagger^2\right)$.

We will select parameters a, b, μ so that $\ddot{R}_k$ matches R_k on the first two moments for all values of ν and $\delta_\dagger$. We can do this using matrix traces to exactly calculate $\sum_{j=k+1}^{n-q} \lambda_j$ and $\sum_{j=k+1}^{n-q} \lambda_j^2$. If we then set the first moments equal we get

$$\mathbb{E}\left[R_k\right] = \nu\left(1+\delta_\dagger^2\right) \sum_{j=k+1}^{n-q} \lambda_j = \nu\left|\lambda_k\right|\left(1+\delta_\dagger^2\right)(a-b+\mu) = \mathbb{E}\left[\ddot{R}_k\right],$$

yielding the constraint

$$a - b + \mu = \left|\lambda_k\right|^{-1} \sum_{j=k+1}^{n-q} \lambda_j =: C_1.$$

Similarly, setting the variances equal we have

$$\mathrm{Var}\left[R_k\right] = 2\nu^2\left(1+2\delta_\dagger^2\right) \sum_{j=k+1}^{n-q} \lambda_j^2 = 2\nu^2\lambda_k^2\left(1+2\delta_\dagger^2\right)(a+b) = \mathrm{Var}\left[\ddot{R}_k\right],$$

which yields the constraint,

$$a + b = \lambda_k^{-2} \sum_{j=k+1}^{n-q} \lambda_j^2 =: C_2.$$

With these constraints, we select a, b, μ as follows

1. If $C_1 \in [-C_2, C_2]$, we set $a = (C_2 + C_1)/2$ and then set $b = C_2 - a$ and $\mu = 0$.

2. If $C_1 > C_2$, we set $a = C_2$, $b = 0$ and $\mu = C_1 - C_2$.

3. If $C_1 < -C_2$, we set $b = C_2$, $a = 0$ and $\mu = C_1 + C_2$.

This specification deliberately minimizes the contribution of μ to the mean relative to a and b while respecting our constraints. As discussed above in Section 2.4, this has the important consequence of ensuring large values of a at loci where the spectrum reaches a plateau (in between recombination hotspots). This large a in turn ensures a reliable p-value estimation at these loci.

Bringing everything together, our final null distribution is

$$\nu\left(\sum_{j=1}^{k^\star} \lambda_j W_j\left(\delta_\star^2\right) + \sum_{j=k^\star+1}^{k} \lambda_j W_j\left(\delta_\dagger^2\right) + |\lambda_k|\left(X_{a,\delta_\dagger^2} - X'_{b,\delta_\dagger^2} + \mu\left(1+\delta_\dagger^2\right)\right)\right). \tag{26}$$

Critically, this parameterization of the null distribution is expressed in two sets of orthogonal parameters. The first set are our spectral parameters $\{\lambda_j\}_{j=1}^{k}$, a, b, and μ corresponding to the spectrum of PMP. The second are our mis-specification parameters $\nu, \delta_\star^2, \delta_\dagger^2$ corresponding to features of the confounding process. This means that, given the phenotype vector Y and the spectral parameters at a given locus, we can readily recompute the observed p-value for that locus for any set of misspecification parameters. Thus, if we see inflation in the Q-Q plot of observed quadratic form p-values for a given phenotype, we can readily adjust the mis-specification parameters and recalculate our p-values in order to correct and calibrate the Q-Q plot.

2.6 Fast Trace Calculation

Here we present our trace calculation approach for a *symmetric* matrix $M \in \mathbb{R}^{n\times n}$. Let $B = I_n - QZ$ where $Q \in \mathbb{R}^{n\times m}$ and $Z \in \mathbb{R}^{m\times n}$. Our goal is to calculate $\|BMB\|_{HS}^2$ and $\operatorname{diag}(BMB)$ efficiently using a set of worker nodes. First we compute $J = ZM \in \mathbb{R}^{m\times n}$ (mn^2 FLOPs). Second we compute $X = Q(JQ) - MQ \in \mathbb{R}^{n\times m}$ ($mn^2 + 2nm^2 + nm$ FLOPs). Then map out the relevant sub-blocks of Q, Z, J, X to each worker as follows:

$$\begin{aligned}(I-QZ)M(I-QZ) &= M - QZM - MQZ + QZMQZ\\ &= M + XZ - QJ\end{aligned}$$

So,

$$\begin{aligned}\|(I-QZ)M(I-QZ)\|_{HS}^2 &= \sum_{i,j}\left(M_{ij} + X_{i\cdot}^T Z_{\cdot j} - Q_{i\cdot}^T J_{\cdot j}\right)^2\\ &= \sum_{i,j}\left(M_{ij} + \sum_{l=1}^{m}\left(X_{il}Z_{lj} - Q_{il}J_{lj}\right)\right)^2\end{aligned}$$

This accumulation requires a total of $(4m+1)n^2$ FLOPs. Since $\operatorname{diag}(BMB)_i = M_{ii} + \sum_{l=1}^{m}(X_{il}Z_{li} - Q_{il}J_{li})$, $\operatorname{diag}(BMB)$ is easily obtained as a by-product of this calculation. Taking the sum of those diagonal elements to obtain $\operatorname{tr}(BMB)$ requires $n-1$ FLOPs. Adding the FLOPs together, we obtain a total of $(6m+1)n^2 + 2nm^2 + nm + n - 1$ FLOPs. Since $m \geq 1$, this is upper bounded by $7mn^2 + 3nm^2$. Connecting this result back to our notation in Section 2.5, this means we can calculate our desired traces η_1 and η_2 with fewer than $7(q+1)n^2 + 3n(q+1)^2$ FLOPs where q is the number of columns in our background covariate matrix A.

If M is distributed in columns across nodes, we can accelerate the above calculation by exporting $W = \begin{bmatrix} X & -Q \end{bmatrix} \in \mathbb{R}^{n\times 2m}$ to every node and the appropriate columns of $\begin{bmatrix} Z \\ J \end{bmatrix} \in \mathbb{R}^{2m\times n}$ to their corresponding nodes.

2.7 Haplotype Simulation

We simulated haplotypes from three 1000 Genomes Populations – Yoruba, Han Chinese, and Central European – using a demographic model adapted from the `msprime` [8] demography tutorial [9], which itself was based on the population parameters related those three populations presented in [10]. We only made one modification to the demographic model specified in [9]: we use the Discrete Time Wright–Fisher model for the first 100 generations into the past before reverting back to the classic Hudson model as proposed in [11]. The mutation rate was set to 1.2×10^{-8}.

Each 1 Mb region of simulated haplotypes was generated using a population-specific human recombination map estimated by `pyrho` [12]. Explicitly, in each simulation, we randomly selected one of our three 1000 Genomes populations (YRI, CHB, or CEU) and 1 Mb segment from genome (excluding heterochromatic regions), then we loaded the recombination map estimated for that population and region by `pyrho`.

Finally, all singleton variants (those with a derived allele count of 1) were removed from the simulated haplotypes before being passed to any association testing method.

2.8 Running ARG-Needle

We ran ARG-Needle using the `arg_association` command line utility. This utility is automatically installed when installing `arg-needle-lib` with `pip` and documented in the ARG-Needle manual available at `https://palamaralab.github.io/software/argneedle/manual/`. As of March 20, 2025, we found that the python `logging` module (available at `https://pypi.org/project/logging/`) imported by `arg_association` has not been updated since 2013. We could not run `arg_association` because the `logging` module was not compatible with the python installation (version 3.12.3) required for our other simulation utilities. Upon inspecting the source code for the `arg_association` utility available on GitHub at `https://github.com/PalamaraLab/arg-needle-scripts/blob/main/arg_needle_lib/arg_mlma/scripts/association.py`, we found that the `logging` module was only used to print progress statements for the user. Commenting out all lines invoking the `logging` module allowed us to successfully run `arg_association`. We saved this modified script as `arg_needle_association_without_logging.py` and used it to perform association testing with the ARG-Needle library.

Since singletons were excluded from all of our simulations, we ran ARG-Needle using `arg_needle_association_without_logging.py` with optional argument `-min-mac 2` so that it would not lose power by attempting to test singletons. We also set the optional argument `-sampling_rate 1e-3`. Otherwise ARG-Needle was run with all default parameters. Filtering the results table returned by ARG-Needle down to rows with `MU_STAR` $\leq 10^{-5}$, we were able to obtain results as if we had invoked ARG-Needle with `-sampling_rate 1e-5`. Similarly, filtering down to rows with `MU_STAR` $\leq 10^{-7}$, we were able to obtain results as if we had invoked ARG-Needle with `-sampling_rate 1e-7`.

2.9 Running ACAT-O

ACAT-O was run using the `STAAR` function from the STAAR R package available at `https://github.com/xihaoli/STAAR`. For each simulation, ACAT-O was run on all genotypes within the causal region. The ACAT-O was run testing rare variants with MAF < 0.01 (by setting `maf_cutoff`=0.01, the default) and all variants (by setting `maf_cutoff`=1). Otherwise, ACAT-O was run with all default settings.

2.10 Calculating Discovery Thresholds

Taking the same approach we used in our power simulations, we ran null simulations to estimate a discovery threshold to control the genome-wide family-wise error rate (FWER) at 0.05 for LOCATER, SMT, AN3, AN5, and AN7. Under the haplotype simulation framework described in the Haplotype Data Simulation subsection of the Methods in the main text, we simlated 900 independent genomic datasets, each consisting of a 1 Mb segment observed for 30,000 indidividuals. For each of these segments, we sampled 10 independent null simulations of the phenotype vector Y as detailed in the Phenotype Simulation subsection of the Methods in the main text. We applied every testing method to each of the resulting 9,000 phenotype vectors and corresponding 1 Mb genomic segments. We stored the smallest p-value obtained the entire segment, yielding 9,000 p-values per method. Since, be default, LOCATER conserves compute by only testing variants with single-marker test p-values smaller than 10^{-4}, sometimes LOCATER did not return any p-value for a given

simulation. This occurred in 3,363 out of our 9,000 simulations. For these simulations, we simply assigned a p-value of 1 to LOCATER.

Since the genome is $3,000$ Mb long and each of the resulting 9,000 p-values was estimated by taking the smallest p-value over a 1 Mb segment, we estimated our 0.05 genome-wide FWER threshold by multiplying the 5th percentile of the resulting 9,000 p-values by $3,000$.

We used the same 9,000 null simulations to estimate a 0.05 genome-wide FWER threshold for ACAT-O (all) and ACAT-O (rare). However, we only ran ACAT-O on the central 10 kb causal window in each simulation rather than over the entire 1 Mb region. If we consider a genome-wide screen using a 10 kb sliding window with 5kb overlap between adjacent windows, that's a total of $600,000$ genome-wide tests. Thus we multiplied the 5th percentile of the resulting 9,000 p-values obtain for ACAT-O (all) and ACAT-O (rare) by $600,000$ to obtain their corresponding discovery thresholds.

2.11 Phenotype Rank Matching

Before we run LOCATER, as has become standard practice in testing quantitative traits, we require that the phenotype residuals be rank normalized after fitting background covariates. The theory underlying SD requires that the phenotypes be independent under H_0^C. Standard inverse-rank-normalization maps phenotype residuals to a fixed grid of values based on the Gaussian CDF, which in turn induces dependency between phenotypes. In order to avoid inducing that dependency, we substitute each phenotype residual with a rank-matched Gaussian random variable. This achieves a rank normalization that is very similar to inverse-rank-normalization but with a small amount of noise added in order to make the normalized phenotypes truly independent under the null hypothesis.

Explicitly, given some raw phenotype vector $\widetilde{Y} \in \mathbb{R}^n$ and a matrix of background covariates $A \in \mathbb{R}^{n \times q}$ (including an intercept), we begin by calculating the residuals $\widetilde{Y}^{\perp} = \widetilde{Y} - A\left(A^{\top}A\right)^{-1}A^{\top}\widetilde{Y}$ of the standard ordinary least squares model

$$\widetilde{Y} \sim A\alpha + \sigma\epsilon. \tag{27}$$

Then we find the permutation $\pi : [n] \to [n]$ that orders the residuals $\widetilde{Y}^{\perp}$ such that $\widetilde{Y}^{\perp}_{\pi(1)} \leq \widetilde{Y}^{\perp}_{\pi(2)} \leq \ldots \leq \widetilde{Y}^{\perp}_{\pi(n)}$. We simulate a new vector of Gaussian phenotypes, $Z \sim N(0, I_n)$ and likewise find the permutation $\pi' : [n] \to [n]$ that orders the entries of Z such that $Z_{\pi'(1)} \leq Z_{\pi'(2)} \leq \ldots \leq Z_{\pi'(n)}$. Then we obtain our normalized vector of rank-matched phenotypes, $Y \in \mathbb{R}^n$, by assigning $Y_j = Z_{\pi'(\pi^{-1}(j))}$.

References

[1] Jacob Cohen. *Statistical power analysis for the behavioral sciences.* Routledge, 2013.

[2] Na Li and Matthew Stephens. Modeling linkage disequilibrium and identifying recombination hotspots using single-nucleotide polymorphism data. *Genetics*, 165(4):2213–2233, 2003. doi: 10.1093/genetics/165.4.2213.

[3] Leo Speidel, Marie Forest, Sinan Shi, and Simon R Myers. A method for genome-wide genealogy estimation for thousands of samples. *Nature Genetics*, 51(9):1321–1329, 2019. doi: 10.1038/s41588-019-0484-x.

[4] Louis J. M. Aslett and Ryan R. Christ. kalis: a modern implementation of the Li & Stephens model for local ancestry inference in R. *BMC Bioinformatics*, 25(1), feb 2024. doi: 10.1186/s12859-024-05688-8.

[5] Vivian Link, Joshua G Schraiber, Caoqi Fan, Bryan Dinh, Nicholas Mancuso, Charleston WK Chiang, and Michael D Edge. Tree-based QTL mapping with expected local genetic relatedness matrices. *The American Journal of Human Genetics*, 110(12):2077–2091, 2023. doi: 10.1016/j.ajhg.2023.10.017.

[6] Andrew C Berry. The accuracy of the gaussian approximation to the sum of independent variates. *Transactions of the American Mathematical Society*, 49(1):122–136, 1941.

[7] Carl-Gustav Esseen. On the liapounoff limit of error in the theory of probability. *Arkiv för Matematik, Astronomi och Fysik.*, A28:1–19, 1942.

[8] Jerome Kelleher, Alison M Etheridge, and Gilean McVean. Efficient coalescent simulation and genealogical analysis for large sample sizes. *PLoS Computational Biology*, 12(5):e1004842, 2016. doi: 10.1371/journal.pcbi.1004842.

[9] tskit Developers. Demography. https://msprime.readthedocs.io/en/stable/tutorial.html#demography, 2020. Accessed: 2020-07-15.

[10] Ryan N Gutenkunst, Ryan D Hernandez, Scott H Williamson, and Carlos D Bustamante. Inferring the joint demographic history of multiple populations from multidimensional SNP frequency data. *PLoS Genetics*, 5(10):e1000695, 2009.

[11] Dominic Nelson, Jerome Kelleher, Aaron P Ragsdale, Claudia Moreau, Gil McVean, and Simon Gravel. Accounting for long-range correlations in genome-wide simulations of large cohorts. *PLoS Genetics*, 16(5):e1008619, 2020.

[12] Jeffrey P Spence and Yun S Song. Inference and analysis of population-specific fine-scale recombination maps across 26 diverse human populations. *Science Advances*, 5(10):eaaw9206, 2019.
